# Influenza-Associated Streptococcus pneumoniae Meningitis: A Complex Clinical Presentation

**DOI:** 10.7759/cureus.84926

**Published:** 2025-05-27

**Authors:** Justine Chinnappan, Rami Al-Handola, Huda Marcus

**Affiliations:** 1 Internal Medicine, Hurley Medical Center - Michigan State University, Flint, USA

**Keywords:** influenza, meningitis, pneumococcal vaccination, streptococcus pneumoniae meningitis, vaccination

## Abstract

This case report underscores the intersection of influenza and Streptococcus pneumoniae, portraying a middle-aged female with multiple risk factors who developed pneumococcal meningitis subsequent to influenza infection. Despite lacking influenza and pneumococcal vaccination, timely diagnosis and appropriate antibiotic therapy led to substantial clinical improvement. The case emphasises the imperative of influenza and pneumococcal vaccination, particularly in at-risk individuals, to avert severe complications like meningitis and sinusitis. It also underscores the necessity of remaining vigilant for secondary bacterial infections in those with influenza-like symptoms, especially amid underlying comorbidities. Swift recognition and management are pivotal in curbing morbidity and mortality associated with influenza-related complications. Emphasising the crucial role of vaccination and timely intervention in improving patient outcomes stands as a paramount consideration.

## Introduction

Influenza, caused by influenza A or B viruses, is a significant acute viral respiratory tract disease, with substantial morbidity and mortality rates. According to CDC data, between 2010 and 2024, influenza contributed to an annual range of 100,000 to 710,000 hospitalizations and 4,900 to 52,000 deaths in the USA, with peak seasons typically occurring during the winter months [1]. To mitigate these risks, the CDC recommends influenza vaccination ideally in September or October. Influenza infection can lead to a spectrum of complications, including secondary bacterial infections, respiratory, cardiovascular, neurological manifestations, and multisystem inflammatory syndrome. One of the most common complications of influenza is bacterial pneumonia, which can occur within days of influenza infection and poses a higher risk, particularly for elderly individuals and those with predisposing conditions [2,3]. Notably, there exists a significant synergism between influenza virus and Streptococcus pneumoniae, leading to heightened mortality rates, especially in cases of pneumonia [3,4]. Post-influenza Streptococcus pneumoniae meningitis is an exceedingly rare yet potentially fatal occurrence. Here, we present a case of a middle-aged female with multiple risk factors, unvaccinated for influenza, who developed Streptococcus pneumoniae meningitis following influenza infection. This case underscores the importance of influenza and pneumococcal vaccination as a preventive measure against severe complications such as meningitis, further emphasising the critical role of vaccination in public health efforts.

## Case presentation

A 62-year-old female presented to the emergency department (ED) during the winter season of December with a one-week history of congestion, cough, rhinorrhoea, and right flank pain. She denied any urinary symptoms. Her medical history included type 2 diabetes mellitus, a previous stroke without residual weakness, and hypertension. There was no history of vaccination against influenza, pneumococcal, or COVID-19 infection. Initial testing confirmed Influenza A infection by reverse-transcriptase polymerase chain reaction (RT-PCR). After being discharged with supportive management as she was outside the anti-influenza treatment window, she returned to the ED one day later with severe right-sided pleuritic chest pain and right flank pain. On initial examination, she was alert and oriented, hemodynamically stable, saturating well in room air, and was afebrile without any costovertebral angle (CVA) tenderness. Approximately nine hours into her observation in the ED, she became febrile, tachycardic, lethargic, confused, and slow to respond. Her Glasgow Coma Scale (GCS) fluctuated between 10 and 13, neurological exam showed no focal neurologic deficit, and no localized weakness. The pulmonary exam was unremarkable with symmetric air entry and no adventitious sounds. The rest of the examination was normal.

Laboratory investigations revealed significantly elevated leukocytes with neutrophilic predominance and lactic acidosis (Table 1). Other blood counts and chemistry profiles were grossly unremarkable. Cerebrospinal fluid (CSF) studies showed neutrophilic pleocytosis, with elevated protein, normal glucose, and a positive PCR for Streptococcus pneumoniae (Table 2).

**Table 1:** Laboratory investigations obtained during initial evaluation HPF: high power field

	VALUES	REFERENCE RANGE
Blood work		
Leukocyte count (neutrophils)	24,800 cells/UL (90%)	4.0 - 10.8 K/UL (36-75%)
Lactate	3.1 mmol/L	0.5 - 2.2 mmol/L
Blood culture	Negative	Negative
Urinalysis and microscopy		
Protein	2+	Negative mg/dL
Blood	2+	Negative
Leukocyte esterase	Trace	Negative
Leukocyte	5-10 cells/HPF	<=5/HPF
Erythrocytes	15-25 cells/HPF	<=2/HPF

**Table 2:** Cerebrospinal fluid (CSF) studies obtained. CSF: Cerebrospinal fluid, PCR: Polymerase Chain Reaction

	VALUES	REFERENCE RANGE
CSF Analysis		
Clarity	Turbid	Clear, Colorless
Protein	228 MG/DL	15 - 45 MG/DL
Glucose	41 MG/DL	40 - 70 MG/DL
Leukocyte count	6,250 cells/CUMM	0 - <10/CUMM
Polymorphic nuclear cells	94%	
Erythrocyte count	100 cells/CUMM	<=1/CUMM
CSF Gram Stain		
Moderate WBCs and Gram-positive cocci in pairs		
CSF Culture		
Negative		
CSF PCR	Positive for Streptococcus pneumoniae	Negative

**Figure 1:**
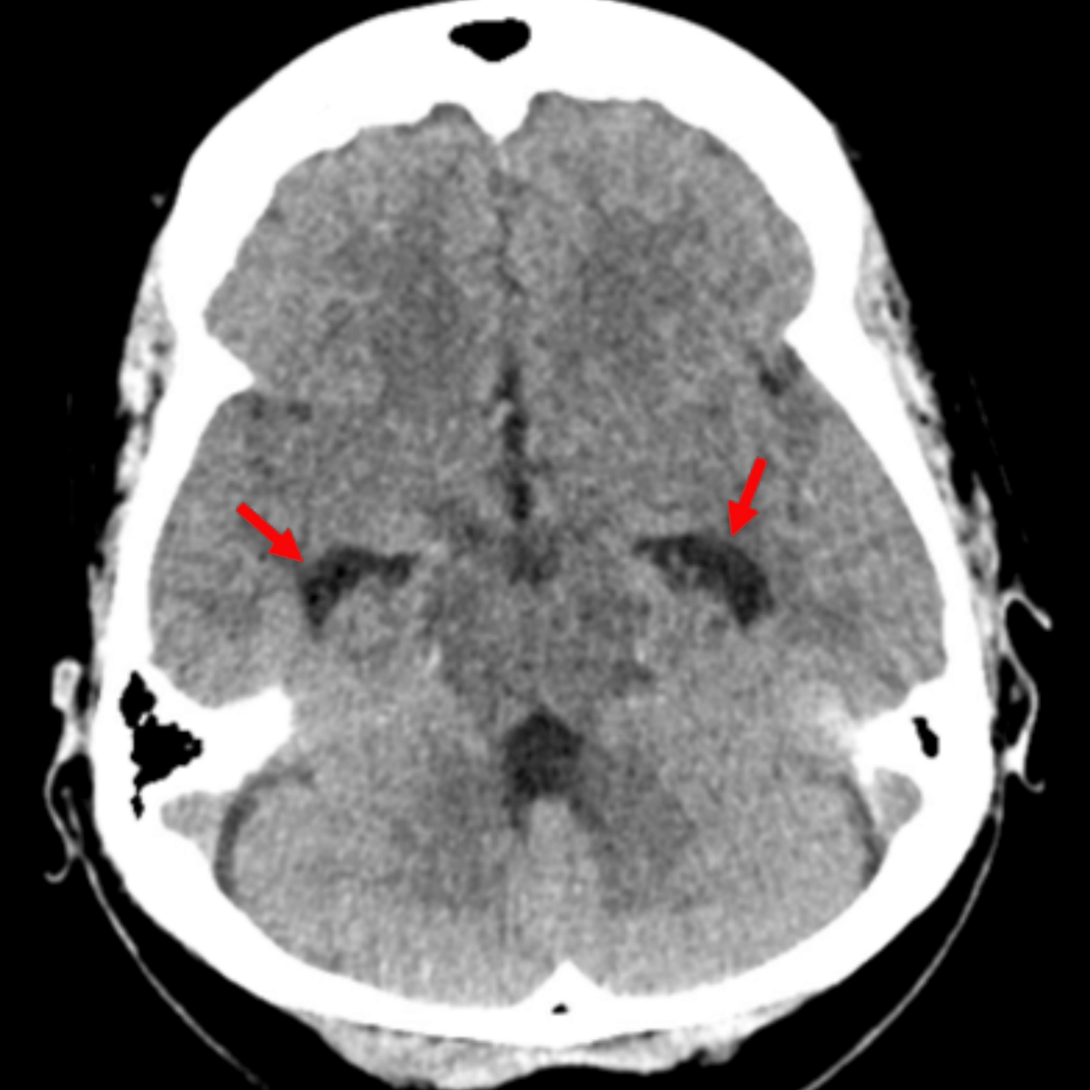
Computed Tomography of the head showing dilated temporal horn (red arrows) of the bilateral lateral ventricles

**Figure 2:**
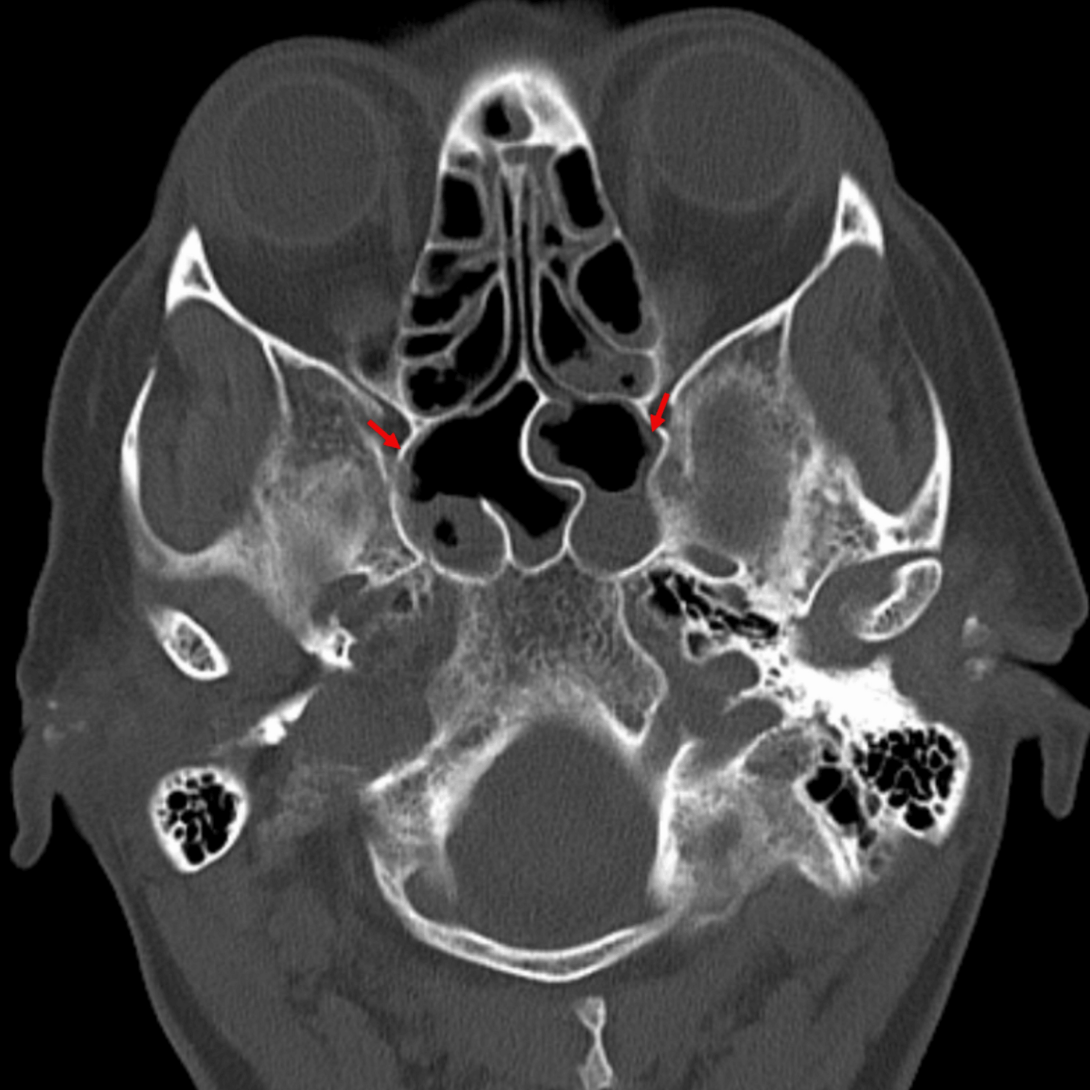
Computed Tomography of the head showing air fluid level in bilateral sphenoid sinus (red arrows)

Computed Tomography Angiogram (CTA) of the chest was negative for pulmonary embolism. Computed Tomography (CT) head without contrast revealed mild dilated temporal horn of both lateral ventricles concerning for hydrocephalus along with opacification of the left maxillary and bilateral sphenoid sinuses (Figures 1, 2). Magnetic Resonance Imaging (MRI) of the head with contrast done two days later revealed resolution of the hydrocephalus with no acute intracranial hemorrhage, mass effect, or abnormal enhancing lesion. It also showed mild mucosal thickening involving bilateral sphenoid, ethmoid, frontal, and maxillary sinuses. Ultrasound of the abdomen revealed bilateral simple renal cysts and an inferior right hepatic lobe cyst.

The patient's presentation with respiratory symptoms, right-sided pleuritic chest pain, altered mental status, and laboratory findings of leukocytosis and abnormal CSF analysis prompted consideration of several differential diagnoses, including post-influenza virus infection pulmonary syndrome, pulmonary embolism, early pyelonephritis, and meningitis or encephalomeningitis. While the recent diagnosis of influenza A infection raised concern for post-influenza virus infection pulmonary syndrome, negative imaging findings on CTA of the chest and the absence of significant respiratory distress ruled out this diagnosis. Similarly, pulmonary embolism was considered but deemed unlikely due to negative CTA findings and the presence of altered mental status. The right flank pain raised suspicion for early pyelonephritis, however, the absence of urinary symptoms and negative imaging findings on ultrasound of the abdomen argued against this diagnosis. Given the patient's altered mental status, leukocytosis, and abnormal CSF analysis, bacterial meningitis and encephalomeningitis were primary considerations. Imaging studies revealed signs of sinusitis, raising the possibility of sinusitis-related complications such as meningitis. The positive PCR for Streptococcus pneumoniae in the CSF confirmed the diagnosis of pneumococcal meningitis. Through systematic evaluation and ruling out of other potential causes, the final diagnosis was established, allowing for the timely initiation of appropriate treatment.

Given the clinical suspicion of meningitis following influenza infection, empiric broad-spectrum antibiotic therapy was initiated, including ampicillin, vancomycin, cefepime, and acyclovir prior to lumbar puncture. Following confirmation of the causative organism by CSF PCR, antibiotic therapy was adjusted to intravenous vancomycin and ceftriaxone. The decision for antibiotic therapy was influenced by the severity of the patient's presentation, the presence of risk factors for complications, and the need for prompt intervention to prevent further deterioration. Dexamethasone was deferred because the patient had already received broad-spectrum antibiotics due to concerns of sepsis, and meningitis was considered only after the patient later developed altered mentation.

She became afebrile with improvement in mentation within 24 hours of antibiotic initiation. During her recovery, she had episodes of headache which were controlled by analgesics. Otherwise, she had an unremarkable recovery. She had excellent clinical recovery and was discharged on day eight of antibiotic treatment to complete the course of ceftriaxone for a total of 14 days as an outpatient through a midline. She was advised to follow up with a neurologist and her primary care physician for reevaluation in one week.

## Discussion

The human respiratory tract harbors a complex microbial community composed of commensal and opportunistic pathogens, the most common being bacteria such as S. aureus and S. pneumonia. Typically, these microbes are part of the commensal flora, but dysbiosis in this community may result in the displacement of opportunistic pathogens to harmful pathogens, resulting in pathogenic invasion. Bacterial infections following influenza are an important cause of morbidity and mortality worldwide. The interaction between influenza virus and Streptococcus pneumoniae, a leading cause of bacterial meningitis and sinusitis, is complex and multifactorial. While the precise mechanisms underlying the facilitation of invasive disease by Streptococcus pneumoniae following influenza coinfection remain the subject of ongoing research, several hypotheses have been proposed. Evidence suggests that pneumococci take advantage of the influenza-mediated increase in host-derived sialylated substrates to promote replication and spread. In the scenario presented, the mechanism behind pneumococcal meningitis likely initiates with the colonization of the nasopharynx by Streptococcus pneumoniae, where it must evade mucosal entrapment and circumvent host immune responses following local activation.

Various animal models and human studies have proven that prior influenza infection increases the pneumococcal colonization by various mechanisms, including enhanced bacterial adherence due to viral neuraminidase activity, and post-viral immunosuppression [5]. Following colonization, invasive disease can result from bloodstream invasion or direct extension from a sinus infection, especially sphenoid sinus. The presence of sphenoid sinusitis in the patient described in this case report raises the possibility of direct extension of the bacterial infection from the paranasal sinuses to the meninges, leading to pneumococcal meningitis though pneumococcal bacteremia resulting in meningitis is still a possibility. Literature search yielded a single reported case of pneumococcal meningitis occurring post-influenza infection, documenting an unvaccinated middle-aged female who presented with altered mental status shortly after a confirmed diagnosis of influenza B infection [6]. Notably, this individual tested positive for Streptococcus pneumoniae in CSF PCR, alongside radiographic evidence of pneumocephalus and features indicative of sinusitis. The clinical course was complicated by multiple seizures, although eventual recovery was achieved with antibiotic therapy. Comparatively, our case exhibits similarities in terms of being unvaccinated despite underlying risk factors and presenting with signs suggestive of sinusitis subsequent to influenza infection, albeit differing in the strain of influenza virus involved. The clinical course was complicated by intractable headache which resolved following antibiotic therapy. The MRI head done a few days later into treatment revealed resolution of hydrocephalus demonstrated on the initial CT head as well. The findings from this case underscore the importance of vigilance for secondary bacterial infections in patients with influenza-like illness, especially those with underlying comorbidities. 

Early recognition, prompt diagnosis, and appropriate management are paramount in reducing the risk of severe complications and improving patient outcomes. Further research is warranted to elucidate the precise mechanisms underlying the interplay between influenza virus and Streptococcus pneumoniae in the pathogenesis of sinusitis and meningitis and to develop targeted interventions for prevention and treatment. By enhancing our understanding of these interactions, we can better inform public health strategies and clinical practice guidelines aimed at mitigating the impact of influenza-related complications and reducing the burden of disease.

## Conclusions

The key takeaways from this case include the understanding that influenza infection can lead to a range of complications. These complications may encompass secondary bacterial infections, as well as respiratory, cardiovascular, neurological manifestations, and multisystem inflammatory syndrome. It is essential for clinicians to maintain a high degree of suspicion for secondary bacterial infections, such as sinusitis and meningitis, in patients presenting with influenza-like symptoms, particularly those with underlying health conditions.

Additionally, healthcare providers should emphasize the importance of receiving the annual influenza vaccination and appropriate pneumococcal vaccination, especially for high-risk individuals. This proactive approach is crucial for reducing the risk of severe complications, including pneumococcal meningitis and sinusitis.
